# Two-stage hepatectomy after autologous CD133+ stem cells administration: a case report

**DOI:** 10.1186/1477-7819-11-192

**Published:** 2013-08-13

**Authors:** Eloisa Franchi, Maria C Canepa, Andrea Peloso, Letizia Barbieri, Laura Briani, Gabor Panyor, Paolo Dionigi, Marcello Maestri

**Affiliations:** 1 Fondazione IRCCS Policlinico San Matteo, and University of Pavia, Viale Camillo Golgi 19, Pavia 27100PV, Italy

**Keywords:** Liver surgery, Portal vein embolization, Stem cells, Liver regeneration, Colon cancer, Liver metastases

## Abstract

Liver resection is the mainstay of treatment for patients with primary and metastatic liver tumors. However, a large majority of patients present for initial medical evaluation with primary and metastatic liver tumors when their cancer is unresectable. Several trials have been undertaken to identify alternative treatments and complementary therapies. In the near future, the field of liver surgery will aim to increase the number of patients that can benefit from resection, since radical removal of the tumor currently provides the sole chance of cure. This paper reports the case of a patient with an advanced colonic cancer in the era of stem cell therapyIn 2011, a 57 years old white Caucasian man with a previous history of non-Hodgkin lymphoma (NHL) was diagnosed with colon cancer and bilobar liver metastases. Following neoadjuvant therapy, the patient was enrolled in a protocol of stem cell administration for liver regeneration. Surgery was initially performed on the primary cancer and left liver lobe. An extended right lobectomy to S1 was then performed after a portal vein embolization (PVE) and stem cell stimulation of the remaining liver. The postoperative course was uneventful and the patient was free of disease after 12 months. Extreme liver resection can provide a safer option and a chance of cure to otherwise unresectable patients when liver regeneration is boosted by PVE and stem cell administration.

## Background

The treatment of choice for both primary and metastatic liver tumors is radical resection [1]. However, up to 45% of patients present for initial medical evaluation when the parenchymal diffusion of cancer requires more surgical resection than is possible. An accepted evaluation of prospective resection is that 30% of the liver must remain in order for its function to be unaffected, or a safer 40% if there is an underlying disease (for example, chronic hepatitis, diabetes) or previous chemotherapy treatment [2].

Portal vein embolization (PVE) has been proposed as a tool to stimulate liver regeneration when prospective surgical resection is over the limit [3]. This technique relies on the portal injection of several agents into the cancer liver lobes. Commonly, patients present with a large tumor in the right liver lobe and a left lobe that is too small for radical resection. Following PVE, the contralateral segments experience a degree of hypertrophy in the range of 15 to 25%, depending on liver status.

When a patient has a tumor that is technically resectable but the remaining liver is small, PVE can stimulate growth, which can be observed until the volume is within the necessary limit. The process is continuous and the remaining tissue will continue to grow. Studies have reported that the liver can take 150 days to develop a volume large enough to allow surgical resection [4]. However, during this time the disease can continue its progression, and patients could die while waiting. Furthermore, PVE does not prevent the tumor growth inside the occluded portal lobe, and there are concerns regarding its potential when diffusion of cancer is faster than expected [5]. Therefore, a procedure is needed to gain volume as swiftly as possible, while minimizing waiting list time to avoid any advantage for the cancer.

Several recent studies have suggested that stem cells play a key role in the field of tissue regeneration [6]. The liver reacts to any lesion by several naturals paths. Thus, several populations of cells cooperate through different pathways, and their role depends on the nature of the stimulus and the extent of damage. A normally functioning liver will undergo a slow turnover of functional mass based on the proliferation of hepatocytes. Adult hepatocytes proliferate after a normal liver resection when the residual parenchyma can tolerate the damage, while supplying the needs of the whole body. When the loss of tissue is greater, the liver seems to have a pool of sleeping cells that have been found mainly in rodents, called ‘oval cells’ [7]. Such cells are relatively undifferentiated and several studies have demonstrated that they can play a role when the normal hepatocytes are not able to repair by normal mechanisms. These emergency cells can possibly differentiate toward the hepatocyte or biliary cell lines. When the liver suffers an extreme insult, both acute and chronic, stem cells can be mobilized from the bone marrow to participate in the repair [8]. These cells are CD34+ and their number grows steadily after extended liver resections [9], while they do not appear after normal abdominal surgery (that is, gastrointestinal or pancreatic operations). Among this population, CD133+ cells are a subset with special liver engraftment potential [10]. The CD133+ phenotype is typical of some cell lines with multipotent differentiation capacity. Recently, CD133+ cells have been proposed to augment liver regeneration after PVE in selected patients [11,12].

This paper presents a case of two-stage hepatectomy of synchronous liver metastases from colon cancer.

## Case presentation

**Figure 1:**
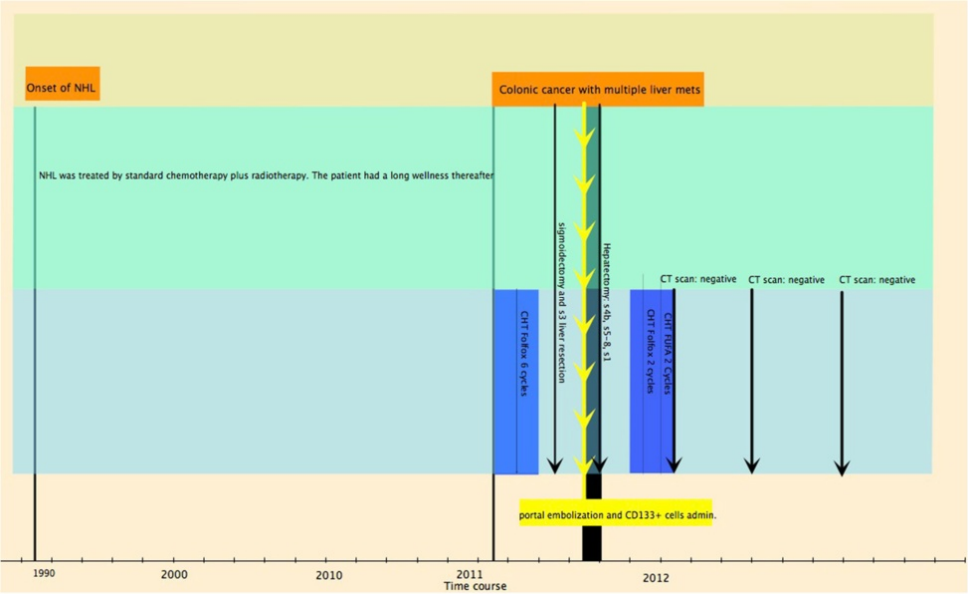
**Timeline of the patient’s course of disease.** After the onset of NHL, the patient had a prolonged period of good health. The colon cancer with synchronous metastases was initially considered unresectable. NHL, non-Hodgkin lymphoma.

A 57 -year-old white Caucasian man was diagnosed with non-Hodgkin lymphoma (NHL) in 1991 (Figure 1). The NHL was treated by a standard protocol of chemotherapy and radiotherapy. The treatment was successful and achieved a complete response. Thereafter, the patient reported good health and normal quality of life. In March 2011, a routine follow-up examination revealed a sigmoid cancer and bilobar multiple liver metastases. In Vittorio Emanuele hospital, Catania, Italy, the oncology team decided to commence a FOLFOX combination chemotherapy regimen. This treatment of six cycles had some success and re-evaluation deemed the disease as stable.

**Figure 2:**
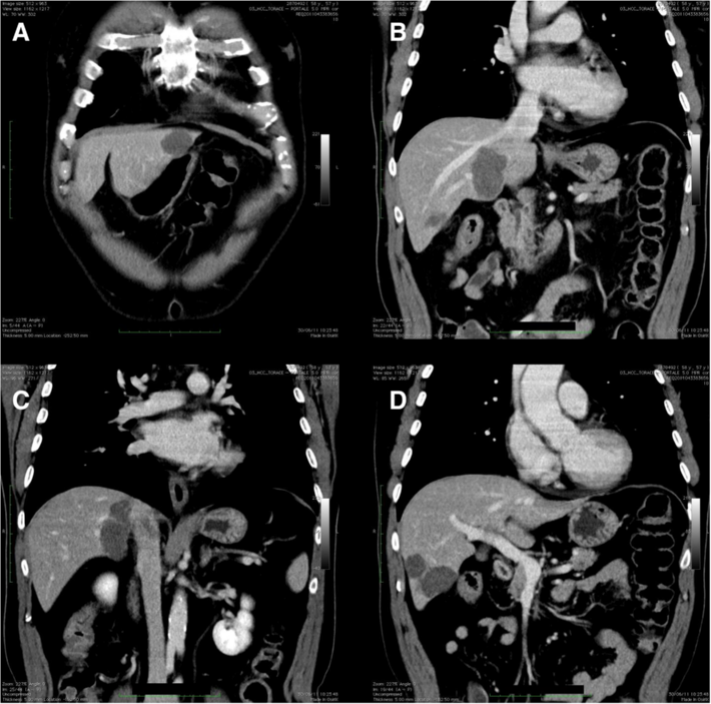
**A CT scan demonstrated advanced bilobar disease. (A)** Cancer nodule in the left lobe and **(B, C, D)** multiple gross deposits in the right lobe of the liver. The tumor also infiltrates S1. CT, computed tomography.

**Figure 3:**
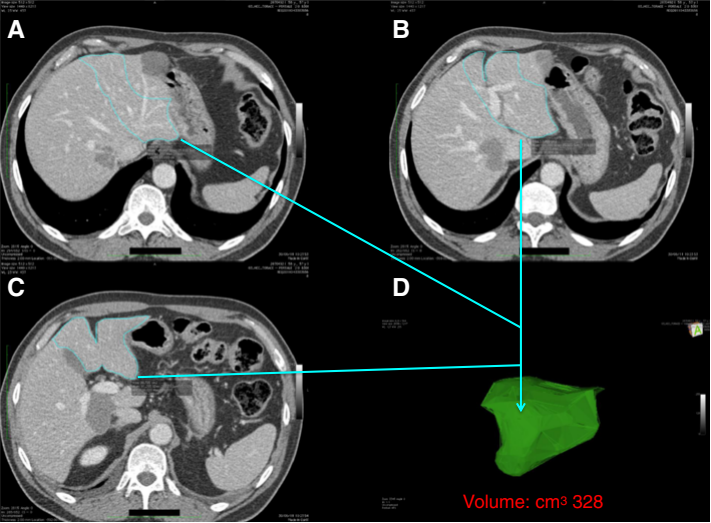
**Volumetric preoperative study. (A, B, C)** The study demonstrated a future liver remnant volume (**D**: FLRV) below the minimum of 40% of total liver volume (TLV). This percentage is generally accepted as the lower limit to allow an extended liver resection with a reasonable probability of success when there is an underlying disease, such as chronic hepatitis, cirrhosis or previous chemotherapy. FLRV, future liver remnant volume; TLV, total liver volume.

The patient was then referred to the liver unit at Fondazione IRCCS Policlinico San Matteo, University Hospital, Pavia, Italy. An additional computed tomography (CT) scan was undertaken to define the extent of the disease (Figures 2 and 3). The multidisciplinary liver team decided the patient should undergo a two-stage resection, with PVE of the right liver lobe after removal of the primary tumor and hepatic segment 3 (S3), because the patient had a future liver remnant volume (FLRV) of <40% (total liver volume (TLV) = 1,281 cm^3^; FLRV = 328 cm^3^; 25.6%). This percentage is regarded as below the limit for liver resection when a patient has chronic disease or has received chemotherapy. Due to the patient’s history of NHL, the chance of undergoing radical surgery remained questionable, and a protocol of boosted regeneration by autologous administration of CD133+ stem cells was proposed. The local ethics committee authorized the procedure as a compassionate treatment and the patient signed a specific consent form.

In July 2011, the patient was admitted to the surgical ward and underwent a combined resection of the colon and liver (S3). An intraoperative ultrasound (IOUS) was performed to confirm the extent of liver disease and map the major biliovascular structures. The pathology confirmed the R0 result on colon cancer and S3, with one positive lymph node out of 14 in the sigmoid specimen. After an uneventful postoperative course, the patient was discharged on the eighth postoperative day.

In August 2011, the patient was readmitted and received granulocyte-colony stimulating factor (G-CSF) at 10 μg/kg of body weight per day to mobilize CD133+ cells from the bone marrow into the blood stream. From the third day of treatment and then daily, a blood sample was taken and CD133+ cells were counted by cytofluorometry. A leukapheresis was performed when the CD133+ cells reached n = 15/μL (on the fifth day of treatment, in this case). The CD133+ cells were purified by immunomagnetic separation (CliniMACS, Miltenyi Biotec Ltd, Surrey, UK). On the same day the right liver was embolized by routine percutaneous technique, the CD133+ suspension was seeded in the prospective remaining left liver parenchyma. The procedure was tolerated quite well and the patient was discharged on the third postoperative day.

**Figure 4:**
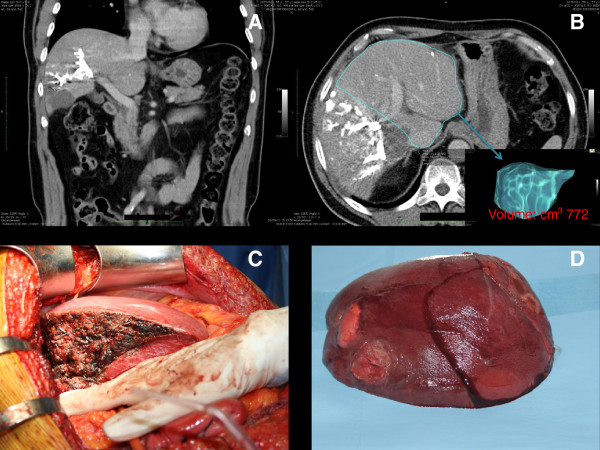
**CT scan, resected liver and specimen. (A, B)** After the portal vein embolization (PVE) and administration of autologous CD133+ cells, the future liver remnant volume (FLRV) reached 772 cm^3^ (15 days after the procedure). The **(C)** resected liver and **(D)** specimen are shown. CT, computed tomography; FLRV, future liver remnant volume; PVE, portal vein embolization.

**Figure 5:**
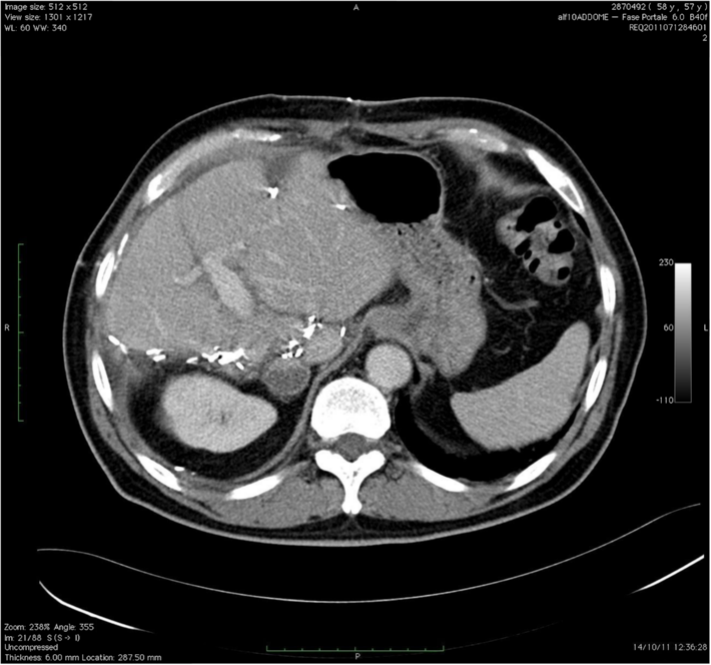
**A CT scan was obtained after surgery.** The outcome was unremarkable with normal liver function. The resected area is highlighted by the hemostatic clips left *in situ*. CT, computed tomography.

Fifteen days later, a volumetric CT scan was undertaken to evaluate the FLRV. The scan demonstrated a growth on the left segment with a FLRV of 772 cm^3^ (60%), which was considered sufficient for radical resection of the tumor burden (Figure 4). A Chevrolet-shaped laparotomy was performed. The IOUS confirmed the tumor in the right lobe with involvement of S1 and part of S4. The liver ligaments were cut and a cava dissection up to the hepatic vein was performed. The hilum was dissected, and the right portal branch, right hepatic artery and bile duct were ligated and sectioned. The right hepatic vein was isolated free at its caval origin and cut between vascular clamps. The stumps were closed with running polypropylene sutures. The parenchyma was transected by cavitron ultrasonic surgical aspirator (CUSA; Valleylab, Boulder, CO, USA). Small vessels were sealed by harmonic scalpel, while those greater than 3 mm were closed by silk ligatures or titanium clips. The resection was extended to the very lateral portion of S4 and to S1 (Figure 5).

The postoperative course was unremarkable and the patient was discharged on the eighth postoperative day. The pathology report demonstrated R0 surgical outcome. Later, the oncology team prescribed a course of chemotherapy (Figure 1). On December 2012, the patient was alive and well, and free from disease at follow-up CT scans.

## Conclusions

Once regarded a risk [13,14], liver surgery is today routine practice for the treatment of several primary and metastatic tumors. However, when the numbers of patients that are deemed eligible for surgery are assessed, a vast majority do not qualify because of anatomy, the number of lesions and amount of FLRV.

PVE is recognized as a tool to stimulate liver regeneration before a liver resection is performed [15]. However, the amount and speed of regeneration can vary with each case, and a number of patients have died while waiting to gain a remaining liver large enough to allow a resection. Recently, stem cells have offered a choice of treatment for several diseases and a possible aid even in the field of liver surgery, with preliminary clinical studies demonstrating a favorable effect when administered after liver damage [16].

We started a clinical study to offer stem cell administration to selected patients. To qualify, the patient must have primary or metastatic liver malignancy, a FLRV below the cutoff for safe resection (<30% or <40% if the patient has an underlying liver condition) and a contraindication to the routine PVE. The patient reported in this case had a previous history of NHL, which was treated by chemotherapy. Thus, the patient’s ability to regenerate after an extended resection was questionable and the case was deemed marginal.

Patients with hematological cancers and metastatic onset of new oncological disease are frequently considered unsuitable for further aggressive surgical treatments. Even with this single case report, the result was appealing and we have begun a local trial on autologous CD133+ stem cells. It must be recognized that there are still concerns about the use of adult stem cells in clinical practice. For example, a patient with cancer can have cancer stem cells in circulation. Thus, we are extensively testing any possible risk of cancer spreading, and to date there has been no evidence that CD133+ autologous administration can negatively affect the biology of the disease. However, this is only a case report and the safety of such procedures must be carefully validated in controlled trials.

While liver regeneration is likely to be boosted by local deposition of specific cells, the question arises about whether regeneration can be any different from the naïve parenchyma. Concerning the hepatic functional reserve, there have been no evident changes after the administration of CD133+ stem cells. These are probably effective in increasing the amount of hepatic parenchyma, but the quality of the regenerated tissue remains the same. Again, this issue requires specific studies which we hope to perform at leading centers. The long-term outcome of this case has not yet been defined and the patient is still undergoing follow-up, but it is important to add that the autologous portal administration of CD133+ cells did not cause any immediate or late adverse effect. Similarly, other reports do not highlight special complications [11].

This report suggests that some cell populations can stimulate hepatic regeneration and their use should be taken into consideration for patients who need extreme liver surgery. We believe that a prospective approach to regenerative medicine in this field will allow an improved indication to resection, while offering a chance of cure to otherwise unresectable patients. Further studies are required to ensure safety and effectiveness of the therapeutic tool against the risk of liver failure after extended resections.

## Consent

Written informed consent was obtained from the patient for publication of this case report and any accompanying images. A copy of the written consent is available for review by the Editor-in-Chief of this journal.

## Abbreviations

CT: Computed tomography; CUSA: Cavitron ultrasonic surgical aspirator; FLRV: Future liver remnant volume; G-CSF: Granulocyte colony-stimulating factor; IOUS: Intraoperative ultrasound; NHL: Non- hodgkin lymphoma; PVE: Portal vein embolization; TLV: Total liver volume.

## Competing interests

The authors declare that they have no competing interests.

## Authors’ contributions

EF and MCC drafted the paper. They were part of the surgical team. AP and LB were in charge of the case and the postoperative care. They were part of the surgical team. LB and GP were responsible to collect the data and to prepare the manuscript. PD was is in charge of the follow-up, prepared the pictures and gave a substantial contribution to the final form of the draft. MM is the first investigator in a clinical trial of stem cells at Fondazione IRCCS Policlinico San Matteo. He performed the operation, designed the stem cell protocol and the postoperative program of therapy. All authors read and approved the final manuscript.
